# SOME WHOS, WHYS, AND HOWS OF BEING MORE DEMENTIA-INCLUSIVE IN AND BEYOND THE CLASSROOM

**DOI:** 10.1093/geroni/igae098.1935

**Published:** 2024-12-31

**Authors:** Joann Montepare

**Affiliations:** Lasell University, Newton, Massachusetts, United States

## Abstract

Demographic statistics predict a dramatic rise in the incidence of dementia (D) and Alzheimer’s disease (AD) as populations age. An estimated 5.3 million Americans were diagnosed with D/AD in 2015, and national predictions are that the number of adults diagnosed with D/AD will rapidly grow each year as the size of the population aged 65 years and older continues to increase (CDC, 2018). As such, researchers, practitioners, and policy makers have called for examining more broadly how we can develop interventions that target stigma, knowledge, diagnosis, resilience, caregiving, and related issues. An important, although overlooked, component of this call is developing educational opportunities for young adults. To this end, this presentation will describe research that aimed to gather information about undergraduate students’ dementia experience, knowledge, and concern. Specifically, a survey of 106 students distributed in diverse classes found that more than 50% of students had experience with someone with D/AD, showed low levels of knowledge, expressed strong concern about developing D/AD, and had an interest in educational activities. As well, this presentation will argue why efforts to be more dementia-inclusive should be incorporated into contemporary age-inclusive initiatives in higher education (Age-Friendly University/AFU, Age Inclusivity Domains of Higher Education/AIDHE). Finally, several activities in (TimeSlips) and beyond the classroom (Dementia Friends) will be described to show how experiential learning opportunities about dementia can be mounted.

